# A barley UDP-glucosyltransferase inactivates nivalenol and provides Fusarium Head Blight resistance in transgenic wheat

**DOI:** 10.1093/jxb/erx109

**Published:** 2017-04-12

**Authors:** Xin Li, Herbert Michlmayr, Wolfgang Schweiger, Alexandra Malachova, Sanghyun Shin, Yadong Huang, Yanhong Dong, Gerlinde Wiesenberger, Susan McCormick, Marc Lemmens, Philipp Fruhmann, Christian Hametner, Franz Berthiller, Gerhard Adam, Gary J. Muehlbauer

**Affiliations:** 1 Department of Plant and Microbial Biology, University of Minnesota, St. Paul, MN 55108, USA; 2 Department of Applied Genetics and Cell Biology, University of Natural Resources and Life Sciences, Vienna, 3430 Tulln, Austria; 3 Department of Agrobiotechnology, IFA-Tulln, Christian Doppler Laboratory for Mycotoxin Metabolism and Center for Analytical Chemistry, University of Natural Resources and Life Sciences, Vienna, 3430 Tulln, Austria; 4 Department of Agronomy and Plant Genetics, University of Minnesota, St. Paul, MN 55108, USA; 5 Department of Plant Pathology, University of Minnesota, St. Paul, MN 55108, USA; 6 USDA-ARS, Mycotoxin Prevention and Applied Microbiology Research Unit, Peoria, IL 61604, USA; 7 Institute for Biotechnology in Plant Production, Department of Agrobiotechnolgy, IFA-Tulln, University of Natural Resources and Life Sciences, Vienna, 3430 Tulln, Austria; 8 Institute of Applied Synthetic Chemistry, Vienna University of Technology, 1060 Vienna, Austria

**Keywords:** *Fusarium graminearum*, Fusarium Head Blight, nivalenol, trichothecene, UDP-glycosyltransferase, wheat.

## Abstract

Fusarium Head Blight is a disease of cereal crops that causes severe yield losses and mycotoxin contamination of grain. The main causal pathogen, *Fusarium graminearum*, produces the trichothecene toxins deoxynivalenol or nivalenol as virulence factors. Nivalenol-producing isolates are most prevalent in Asia but co-exist with deoxynivalenol producers in lower frequency in North America and Europe. Previous studies identified a barley UDP-glucosyltransferase, HvUGT13248, that efficiently detoxifies deoxynivalenol, and when expressed in transgenic wheat results in high levels of type II resistance against deoxynivalenol-producing *F. graminearum*. Here we show that HvUGT13248 is also capable of converting nivalenol into the non-toxic nivalenol-3-*O*-β-d-glucoside. We describe the enzymatic preparation of a nivalenol-glucoside standard and its use in development of an analytical method to detect the nivalenol-glucoside conjugate. Recombinant *Escherichia coli* expressing HvUGT13248 glycosylates nivalenol more efficiently than deoxynivalenol. Overexpression in yeast, *Arabidopsis thaliana*, and wheat leads to increased nivalenol resistance. Increased ability to convert nivalenol to nivalenol-glucoside was observed in transgenic wheat, which also exhibits type II resistance to a nivalenol-producing *F. graminearum* strain. Our results demonstrate the HvUGT13248 can act to detoxify deoxynivalenol and nivalenol and provide resistance to deoxynivalenol- and nivalenol-producing *Fusarium*.

## Highlight

Barley HvUGT13248 catalyzes conversion of nivalenol (NIV) to its non-toxic form 3-*O*-glucoside, and in transgenic wheat confers resistance to NIV-producing *Fusarium graminearum*

## Introduction

Small grain cereals such as wheat and barley are commonly infected by ascomyceteous fungi of the genus *Fusarium*, causing Fusarium Head Blight (FHB), a plant disease which leads to severe yield losses worldwide and contamination with trichothecene mycotoxins (Goswami and Kistler, 2004; Kazan *et al.*, 2012). Trichothecenes are heat-stable sesquiterpenoid compounds routinely found in grain intended for food and feed use (Gottschalk *et al.*, 2009; Streit *et al.*, 2012), constituting a threat to the health of humans and livestock. Trichothecenes are potent inhibitors of protein biosynthesis in eukaryotic cells and act as virulence factors of FHB disease development (Bai *et al.*, 2002; Jansen *et al.*, 2005; McCormick *et al.*, 2011). Inability to produce trichothecenes in *F. graminearum* results in strongly reduced disease severity and spread (Proctor *et al.*, 1995; Bai *et al.*, 2002). In turn, successful detoxification of deoxynivalenol is associated with resistance to disease spread within spikes (Lemmens *et al.*, 2005; Li *et al.*, 2015), which is defined as type II resistance (Mesterhazy, 1995).

The *Fusarium* species causing FHB on wheat and barley produce mainly type A or type B trichothecenes, with the type B trichothecenes deoxynivalenol (DON) and nivalenol (NIV) being the greatest concern in wheat- and barley-growing regions (Alexander *et al.*, 2011). NIV and DON differ only by an additional hydroxyl group at the C4 position in NIV (McCormick *et al.*, 2011). NIV is most prevalent in harvested materials from Asia and South America, whereas DON occurs predominantly in North America and Europe (van der Lee *et al.*, 2015). It is noteworthy that strains producing DON, NIV, and their acetylated derivatives can all be found in the same region (van der Lee *et al.*, 2015), and shifts in chemotype frequency occur and may take place rapidly. For example, a Canadian survey reported a dramatic shift from 15ADON (15-acetyldeoxynivalenol) chemotypes to 3ADON (3-acetyldeoxynivalenol) chemotypes within only 6 years (Ward *et al.*, 2008). Thus, developing germplasm that exhibits resistance to a wide range of trichothecene mycotoxins should provide broad spectrum resistance and reduce the chance of the pathogen undergoing a population shift to a different chemotype composition.

NIV orally ingested by animals is more toxic than DON (Ryu *et al.*, 1988). The European Food Safety Administration established a lower tolerated daily intake (TDI) of 0.7 µg kg^–1^ body weight for NIV, compared with 1 µg kg^–1^ for DON (EFSA CONTAM Panel, 2013). The Food Safety Commission in Japan established a TDI for NIV of 0.4 µg kg^–1^ body weight per day (Food Safety Commission of Japan, 2010). NIV is not a major problem in the USA currently, but NIV-producing *F. graminearum* strains have been discovered (Gale *et al.*, 2011). In contrast to DON, the United States Food and Drug Administration has not established advisory guidelines for NIV (United States Food and Drug Administration, 2010).

DON resistance can be achieved in plants by the enzymatic conversion of the toxin into the non-toxic DON-3-*O*-glucoside (D3G) by substrate-specific UDP-glycosyltransferase (UGT) as first demonstrated in *Arabidopsis thaliana* (Poppenberger *et al.*, 2003). Functional homologs of DON-inactivating UGTs in the agronomically relevant *Pooideae* subfamily have been identified in *Brachypodium distachyon*, rice, and barley (Schweiger *et al.*, 2010, 2013; Pasquet *et al.*, 2016). Among these, the highly DON- and *F. graminearum*-inducible barley *HvUGT13248* gene (Gardiner *et al.*, 2010) provided DON resistance when expressed in *A. thaliana* (Shin *et al.*, 2012), and resistance to DON and DON-producing *F. graminearum* strains in transgenic wheat (Li *et al.*, 2015). The kinetic properties and crystal structure of the *Escherichia coli*-expressed and affinity-purified gene product of *OsUGT79*, a rice gene highly similar to *HvUGT13248*, have been recently described (Michlmayr *et al.*, 2015; Wetterhorn *et al.*, 2016).

Recently, NIV-3-*O*-glucoside (NIV3G) has been described to occur in naturally *Fusarium*-infected barley and other cereals from Finland (Nathanail *et al.*, 2015). The formation of a NIV-glucoside has been demonstrated in NIV-treated wheat (Nakagawa *et al.*, 2011; Yoshinari *et al.*, 2014), and NIV3G was purified from such material. Glycosyltransferases with specificity for NIV therefore exist in different cereal species, and may play a role in defense against NIV producers.

The aim of this study was to determine whether *HvUGT13248* can inactivate and provide resistance to NIV. We show that expression of *HvUGT13248* in yeast and *A. thaliana* confers NIV resistance in both model systems and that NIV3G can be efficiently produced by enzymatic synthesis. Stable transgenic events of the susceptible wheat cultivar ‘Bobwhite’ expressing *HvUGT13248* confer increased ability to detoxify NIV and increased type II resistance to NIV-producing *Fusarium* strains.

## Materials and methods

### Heterologous expression of UGT in yeast

YZGA515 is a toxin-sensitive yeast strain in which three pleiotropic drug resistance genes and a trichothecene-3-*O*-acetyltransferase have been disrupted (*MATa leu2Δ1 trp1Δ63 ura3-52 his3Δ200 lys2-801 ade2-101 pdr5::TRP1 pdr10::hisG pdr15::loxP-KAN*^*R*^*-loxP ayt1::URA3*), effectively rendering the strain unable to grow on low trichothecene concentrations (Schweiger *et al.*, 2010). The strain was transformed with the previously reported UGT genes *DOGT1=AtUGT73C5* (pBP868) (Poppenberger *et al.*, 2003) and *HvUGT13248* (pWS1921) (Schweiger *et al.*, 2010), and the empty vector pYAK7 (pBP910; Poppenberger *et al.*, 2003). These UGTs are under control of the strong constitutive pADH1 promotor and are N-terminally fused to a c-Myc epitope tag. Transformants were selected on synthetic complete medium lacking leucine. Stock solutions of 10 g l^–1^ of DON and NIV were prepared from crystallized toxins, and YPD plates containing 0, 40, 80, and 120 mg l^–1^ of either toxin were prepared. Exponentially grown yeast strains were rediluted in fresh selective medium to OD 0.05 and 0.005, and 3 µl of suspension cultures harboring either of the three constructs were spotted on plates containing increasing concentrations of NIV and DON.

### Recombinant expression and purification of HvUGT13248

HvUGT13248 was expressed in *E. coli* as a fusion protein with an N-terminal His_6_-tag and a maltose-binding protein (nHis_6_-MalE-*HvUGT13248*). The cDNA was amplified from pWS1921 with the oligonucleotide primers 5'-GATATACATATG GCTGTCCACGACG-3' and 5'-TATATAAAGCTTTCAGCT GGCCTGGATGTC-3' and ligated to pCA02 [a derivative of the pET31b-based pKLD116 (Rocco *et al.*, 2008) containing the pET21d multiple cloning site] using *Nde*I and *Hin*dIII (restrictions sites on primers are underlined). nHis_6_-MalE-*HvUGT13248* was expressed with *E. coli* SHuffle^®^ T7 Express lysY (New England Biolabs, Frankfurt am Main, Germany).

Protein expression and purification by immobilized metal ion chromatography (IMAC) on Ni^2+^-charged 5 ml HisTrap Crude FF columns (GE Healthcare, Chalfont St Giles, UK) were performed as recently described for OsUGT79 (Michlmayr *et al.*, 2015). After IMAC, the buffer was changed to 50 mM potassium phosphate pH 7 + 50 mM NaCl + 10% glycerol by gel filtration on Sephadex G25 (GE Healthcare). One-step purified protein was stored in this buffer at −80 °C. Protein concentrations were determined with the Bio-Rad (Hercules, CA, USA) protein assay based on the dye-binding method of Bradford.

Enzyme assays were performed in 100 mM Tris, pH 7 at 37 °C, 2 min reaction time. For kinetic analyses, substrate concentrations ranged from 0.2 mM to 8 mM (NIV) and 0.3 mM to 25 mM (DON). The assays were stopped by transferring 20 μl of sample to 180 μl of methanol. After centrifugation (20 000 g, 5 min) to remove precipitated protein, the samples were further diluted in H_2_O to an expected concentration range of 1 mg l^−1^. The concentrations of NIV/NIV3G and DON/D3G were determined by liquid chromatography coupled with tandem mass spectrometry (LC-MS/MS, see below). Data regression (Michaelis–Menten equation) was performed with SigmaPlot 11.0 (Systat Software, San Jose, CA, USA). Enzyme activity is reported in μmol min^−1^ mg^−1^, which refers to the formation of NIV3G or D3G per mg of protein.

### Synthesis and purification of a NIV-3-*O*-β-d-glucoside standard

NIV3G was produced with OsUGT79 (Michlmayr *et al.*, 2015) in a 44 ml batch containing 1.5 mM NIV, 2.6 mM UDP-glucose, and 0.7 mg ml^–1^ nHis_6_-MalE-*Os*UGT79. The reaction was carried out in 100 mM Tris–HCl pH 7 at 25 °C for 24 h. Purification of NIV3G was carried out using an 1100 series preparative HPLC system equipped with an automatic fraction collector and a multiple wavelength detector (MWD) (all Agilent Technologies, Waldbronn, Germany). A Gemini NX column (150 × 21.2 mm, 5 μm, Phenomenex, Aschaffenburg, Germany) and gradient elution (eluent A, water; eluent B, methanol) was used for the separation of NIV3G from residual glucose and other impurities. The initial condition of 10% B was maintained for 2 min, followed by a linear increase to 60% B within 4 min and to 100% B within 0.1 min. Following a hold time of 1 min at 100%, the initial conditions were achieved with a fast switch to 10% B and the column was equilibrated prior to the next injection. The flow rate was 20 ml min^−1^ and the injection volume was set to 900 μl. The fractions were collected from 4 min to 6 min with the maximum peak duration of 1.5 min using threshold working mode. The collected fractions were pooled, the organic phase was evaporated on a rotary evaporator at 30 °C, and the remaining water phase was removed by lyophilization. The NIV3G crystals were weighed in a glass vial on a microbalance (16 mg) and stored at −20 °C.

### NMR spectroscopy of NIV-3-*O*-β-d-glucoside

^1^H and ^13^C spectra were recorded on a Bruker Avance DRX-400 MHz spectrometer (Bruker, Germany). Data were recorded and evaluated using TOPSPIN 1.3 and TOPSPIN 3.2 (Bruker Topspin, Germany). All chemical shifts are given in ppm relative to tetramethylsilane. The calibration was done using residual solvent signals. Multiplicities are abbreviated as s (singlet), d (doublet), t (triplet), q (quartet), and b (broad signal). Deuterated methanol was purchased from Eurisotop (Gif sur Yvette Cedex, Paris, France).

### Translation assays

To determine the *in vitro* toxicity of DON, NIV, and NIV3G, commercial *in vitro* transcription/translation systems [TnT^®^ T7 Coupled Wheat Germ Extract System and TnT^®^ T7 Coupled Reticulocyte Lysate System (Promega, Madison, WI, USA)] were used. Transcription/translation reactions were performed as described in Varga *et al.* (2015) with one minor change being that reactions with rabbit reticulocyte lysate were stopped after 20 min (instead of 24 min) reaction time. At least three independent assays using individual dilutions were performed for each substance.

### Quantitative determination using LC-MS/MS

LC-MS/MS analysis was performed on a QTrap 4000 mass spectrometer (Sciex, Foster City, CA, USA). Chromatographic separation was achieved on a Gemini C18 (150 × 4.6 mm, 5 µm; Phenomenex, Aschaffenburg, Germany) at 25 °C with a flow rate of 0.8 ml min^–1^. The following water–methanol gradient (eluent A, 80:20, v/v; eluent B, 3:97, v/v; both containing 5 mM ammonium acetate) was used: initial conditions at 0% B were held for 1 min, followed by a linear increase to 50% B within 5 min and with a jump to 100% B. After holding 100% B for 2 min, a fast switch to the initial conditions was performed followed by column equilibration until 10 min. Negative electrospray ionization mode with the following source settings: temperature 550 °C, ion spray voltage 4 kV, curtain gas 30 psi (207 kPa of 99.5% nitrogen), source gas one and two both 50 psi (345 kPa of zero grade air), and collision gas (nitrogen) set to high. For quantitation, two selected reaction monitoring transitions per compound were acquired with a dwell time of 25 ms. The acetate adducts of the analytes (*m/z* 355.1 for DON, *m/z* 371.1 for NIV, *m/z* 517.3 for DON3G, and *m/z* 533.1 for NIV3G) were chosen as precursors, and the declustering potential (DP) was –40 V for DON and NIV, –50 V for DON3G, and –60 V for NIV3G. The following product ions were chosen as quantifier and qualifier, respectively: for DON *m/z* 59.2 [collision energy (CE) of –40 V] and *m/z* 265.2 (CE –22 V), for NIV *m/z* 59.1 and 281.1 (both CE –38 V), for DON3G *m/z* 427.1 (CE –30 V) and *m/z* 59.1 (CE -85 V), and for NIV3G *m/z* 263.0 (CE –30 V) and *m/z* 443.0 (CE –26 V).

### Transgenic *Arabidopsis thaliana* root growth assay

*Arabidopsis thaliana* seeds (Shin *et al.*, 2012) were surface sterilized in a 20% bleach, 0.02% Triton X-100, 0.5% sucrose solution for 10 min with continuous shaking, then washed twice using sterilized distilled water, followed by cold treatment in 4 °C for 3 d in the dark. Sterilized seeds were plated on half-strength Murashige and Skoog (MS) medium (2.15 g l^–1^ MS salt, 0.5 g l^–1^ MES, 0.5% sucrose, and 10 g l^–1^ agar) containing varying amounts of NIV.

The square Petri dishes were positioned vertically at room temperature under 16 h light and 8 h dark periods. Pictures of the plates were taken every 24 h starting from 4 d after germination. The longest root of each seedling was measured using ImageJ Software (Schneider *et al.*, 2012).

### Greenhouse disease testing of transgenic wheat expressing *HvUGT13248* with NIV-producing *F. graminearum*

Seeds from each wheat genotype (Li *et al.*, 2015) were planted into Sunshine MVP growth medium (Sun Gro Horticulture, Agawam, MA, USA) in 6 inch square plastic pots in a greenhouse. A total of 20–32 seeds were planted for each transgenic event with each pot containing four seeds. Twenty seeds of non-transformed controls (‘Bobwhite’, ‘Sumai 3’, and ‘Wheaton’) were also planted at four seeds per pot. Plants were fertilized with one teaspoon of Osmocote (14-14-14 N-P-K, Scotts Company, Marysville, OH, USA) fertilizer per pot at the three-leaf stage. Plants expressing the transgene were detected based on ELISAs (Agdia Inc., Elkhart, IN, USA) with an NPTII antibody as described in Li *et al.* (2015). Only transgenic plants expressing NPTII were analyzed further.

At anthesis, one ﬂoret of a central spikelet of the main spike was inoculated with 10 µl of macroconidial suspension (10^5^ macroconidia ml^–1^ and 0.01% Triton X-100) of the NIV-producing *F. graminearum* strain 02-15 (Gale *et al.*, 2011). Inoculated spikes were covered with transparent plastic bags for 3 d. FHB disease severity was determined as the percentage of spikelets with disease symptoms on the inoculated spikes at 21 d after inoculation. For statistical analysis, Student’s *t*-tests were used to compare each transgenic line with the non-transformed ‘Bobwhite’ control.

DON, NIV, and ergosterol were measured on NIV-producing *F. graminearum*-inoculated wheat via GC-MS as described (Dong *et al.*, 2006; Jiang *et al.*, 2006) using whole spikes sampled at 21 d after point inoculation. Nine spikes were sampled from each genotype of wheat tested in the autumn 2013 greenhouse screen.

### Conversion of NIV to NIV3G *in planta* in transgenic wheat expressing *HvUGT13248*

Transgenic wheat expressing *HvUGT13248*, *HvUGT13248*-#19, and the non-transformed ‘Bobwhite’ control were inoculated at anthesis with 10 µl of aqueous NIV solution (2 µg µl^–1^) between the palea and lemma in the central four florets on the main spike of each plant. In this manner, each spike received 80 µg of NIV. Spikes were sampled at 0, 2, 6, 12, 24, 36, 48, 72, and 96 h, and 14 d after treatment. Nine biological replications were completely randomized during growth and one central spike per replication was used for each time point for each genotype. The four inoculated florets with rachis tissue for each replication were ground in liquid nitrogen, and metabolites were extracted in 4 vols of extraction solvent (50% methanol). NIV and NIV3G levels were ascertained by LC-MS/MS as described above.

## Results

### *HvUGT13248* provides resistance to NIV in yeast

**Fig. 1.**
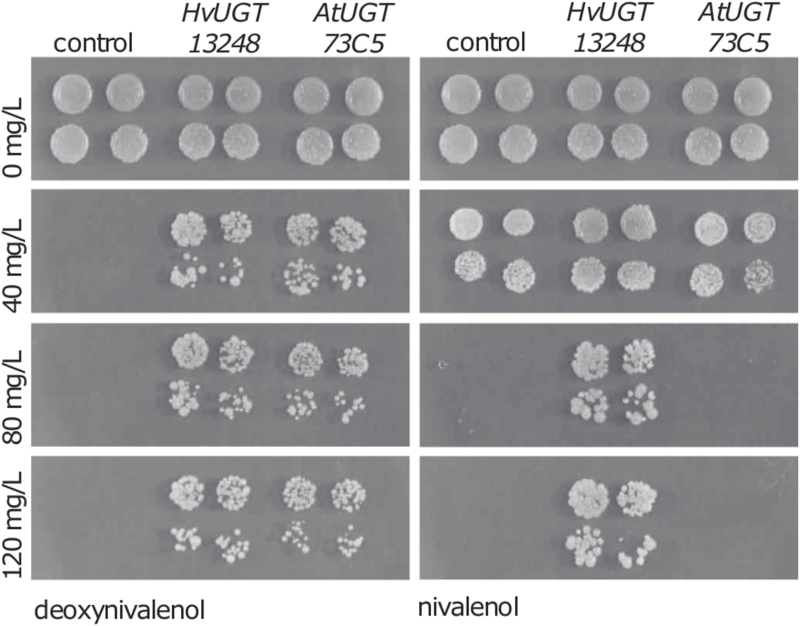
Growth of glycosyltransferase-expressing yeast on rich medium containing the indicated concentrations of DON and NIV.

The glycosylation of DON by plant UGTs is well established as a major detoxification process and can be monitored by the increased accumulation of D3G in plant extracts (Shin *et al.*, 2012; Li *et al.*, 2015; Pasquet *et al.*, 2016). Recently, NIV3G has been detected in barley and other cereal species (Nathanail *et al.*, 2015), which may be due to the activity of either a UGT or different enzymes. Toxin-sensitive baker’s yeast transformed with either the barley *HvUGT13248* or the *A. thaliana AtUGT73C5* gene produce the recombinant proteins, as previously confirmed by western blotting using an antibody detecting the N-terminal c-Myc epitope tag (Schweiger *et al.*, 2010). To test for resistance to DON and NIV, transformants expressing *HvUGT13248* or *AtUGT73C5* were spotted on agar medium containing increasing amounts of NIV or DON. While both UGTs conferred DON resistance as described before, only the transformants expressing *HvUGT13248* grew on plates containing up to 120 mg l^–1^ NIV (Fig. 1). The results also show that NIV is less toxic for yeast than DON. While 40 mg l^–1^ DON was completely inhibitory for the strain containing the empty vector, 40 mg l^–1^ NIV caused only a moderate reduction of growth compared with the control without toxin.

### NIV is converted to NIV-3*-O*-β-d-glucoside by purified HvUGT13248

To investigate the fate of NIV *in vitro*, we examined the biochemical properties of the HvUGT13248 enzyme towards NIV as a substrate. We expressed HvUGT13248 and a previously characterized highly similar rice UGT, OsUGT79, which also converts DON to D3G in *E. coli* (Michlmayr *et al.*, 2015). Both enzymes were produced as fusion proteins with an N-terminal His_6_-tag and a *malE* domain to improve solubility. While OsUGT79 was highly expressed and easily purified, HvUGT13248 was only very weakly expressed and highly unstable, preventing further purification after the first IMAC purification step. Nevertheless, the analysis of the enzymatic reaction by HPLC-MS showed that both OsUGT79 and HvUGT13248 can metabolize NIV into a substance with a mass expected for a NIV-glucoside. HvUGT13248 yielded a product with an identical MS/MS fragmentation pattern to the product of OsUGT79, demonstrating that both generate the same substance.

For structural elucidation of this glucoside, we therefore employed OsUGT79 to synthesize the compound in preparative amounts. From 20 mg of NIV in a first experiment, 16 mg of NIV3G were purified. The NIV-glucoside from this first reaction was purified by preparative HPLC and subjected to NMR analysis. Theoretically, the glucose could be linked to any of the four hydroxy groups in NIV (C3-OH, C4-OH, C7-OH, or C15-OH). The NMR results confirmed exclusive glucosylation of NIV at the C3 position with glucopyranose linked in β-configuration (see Supplementary Fig. S1 at *JXB* online).

Both OsUGT79 and HvUGT13248 recombinant fusion proteins were characterized with respect to kinetic properties towards NIV and DON (Table 1). The *k*_cat_ values for HvUGT13248 might be underestimated, due to lower purity after only one purification step. The results nevertheless indicate that HvUGT13248 has higher affinity and ~5-fold higher catalytic efficiency towards NIV compared with DON.

**Table 1.** Biochemical characterization of recombinant UDP-glucosyltransferases from rice (nHis_6_-MalE-OsUGT79) and barley (nHis_6_-MalE-HvUGT13248).

Catalyst	Substrate	*K*_M_ (mM)	*V*_max_ (µmol min^–1^ mg^–1^)	*k*_cat_	*k*_cat/_ *K*_M_
HvUGT13248	Deoxynivalenol	3.0 ± 0.6	0.49 ± 0.04	>0.78	>0.26
	Nivalenol	1.2 ± 0.3	0.96 ± 0.06	>1.5	>1.3
OsUGT79	Deoxynivalenol^*a*^	0.23 ± 0.06	0.36 ± 0.02	0.57	2.5
	Nivalenol	0.35 ± 0.04	0.38 ± 0.01	0.60	1.7

The displayed values are the results of three independent measurements ±SDs.

^*a*^ Reported in Michlmayr *et al.* (2015).

### NIV3G has minimal inhibitory activity at the ribosomal target

**Fig. 2.**
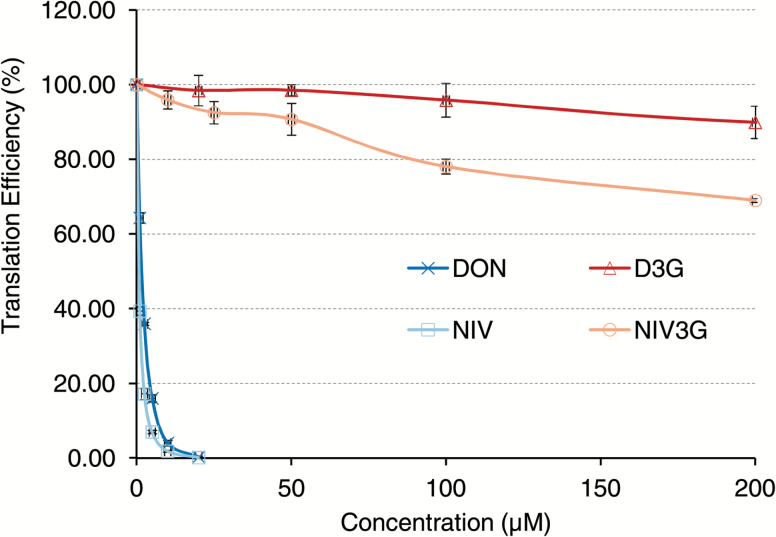
NIV-3-glucoside is less toxic than NIV and DON on wheat ribosomes.

The primary mode of action of trichothecenes is inhibition of eukaryotic protein synthesis. To test whether NIV3G can inhibit animal and plant ribosomes, we utilized coupled *in vitro* transcription and translation systems, where ribosomes either from rabbit reticulocytes or from a wheat germ extract translate a luciferase reporter gene. As shown in Fig. 2 and Supplementary Fig. S2, both NIV and DON efficiently block translation in both systems. For wheat ribosomes, the IC_50_ for DON was 1.6 µM, and 0.7 µM for NIV. Likewise, in rabbit reticulocytes, the IC_50_ values for DON and NIV were 1.4 µM and 0.8 µM, respectively. Thus, NIV is slightly more inhibitory for ribosomes than DON for both animal and plant ribosomes. NIV-glucoside showed strongly reduced inhibitory activity (Fig. 2; Supplementary Fig. S2) in both systems, as previously described for D3G (Poppenberger *et al.*, 2003). At the highest NIV3G concentration (200 µM) used in the translation assays, ~30% inhibition was observed in the wheat germ assay (Fig. 2). The IC_20_ values for NIV and NIV3G were reached at 0.3 µM and 90 µM, respectively, in the wheat germ assay, corresponding to an ~300-fold reduction in translation inhibition. Similarly, in the rabbit reticulocyte system, 600-fold more NIV3G than NIV is required to result in 20% translation inhibition (Supplementary Fig. S2). Results of molecular modeling (Pierron *et al.*, 2016) suggest that the addition of the bulky glucose group should completely prevent interaction with the ribosomal binding site. The slight translation inhibition at extremely high NIV3G levels (200 µM or ~95 mg l^–1^) could theoretically be caused by partial hydrolysis of the glucoside during the assay, as previously observed for acetylated trichothecene (NX-2; Varga *et al.*, 2015). We therefore analyzed samples from the end-points of the assay (stopped by precipitation with acetonitrile). However, no NIV was detectable in any of the samples (data not shown). We can therefore exclude that the minor residual inhibitory effect was due to hydrolytic cleavage of NIV3G.

### Increased NIV resistance in transgenic *A. thaliana* expressing *HvUGT13248*

**Fig. 3.**
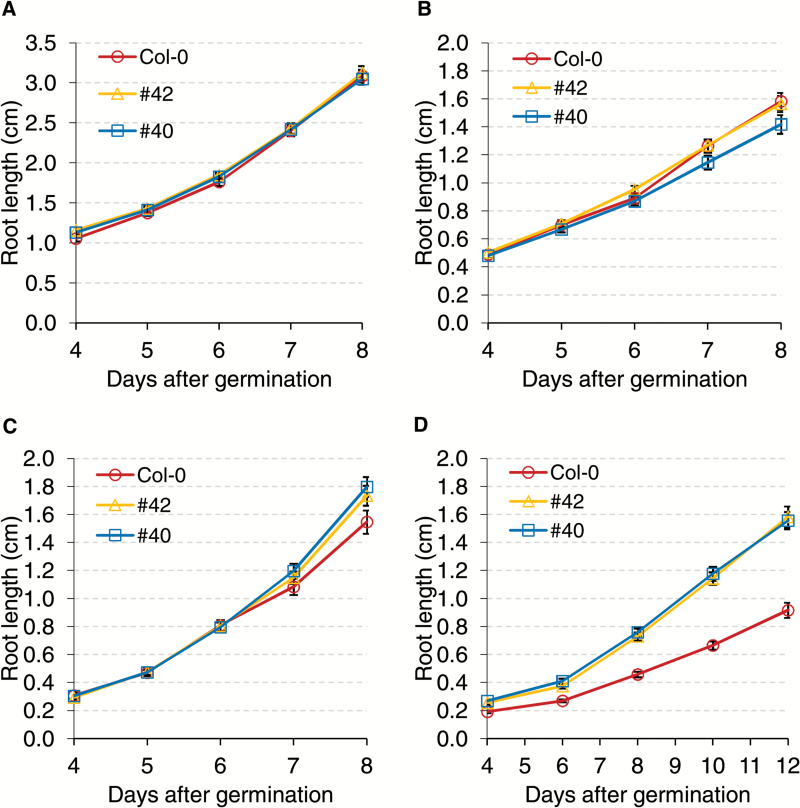
Root growth of transgenic *Arabidopsis thaliana* expressing *HvUGT13248* in the Col-0 background on half-strength MS medium containing (A) 0 mg l^–1^ NIV, (B) 20 mg l^–1^ NIV, (C) 40 mg l^–1^ NIV, and (D) 100 mg l^–1^ NIV.

To investigate whether *HvUGT13248* provides resistance to NIV in plants, we tested transgenic *A. thaliana* expressing *HvUGT13248* on NIV-supplemented growth medium (Fig. 3; Supplementary Fig. S3). Two transgenic lines (#40 and #42) previously reported by Shin *et al.* (2012), along with a non-transformed Col-0 control, were grown on half-strength MS medium supplemented with 0 mg l^–1^ to 100 mg l^–1^ NIV. The results show that in the control treatment (0 mg l^–1^ NIV), root lengths of the transgenic lines were not significantly different from the non-transformed Col-0 (Fig. 3A: Supplementary Fig. S3). When grown on medium containing 100 mg l^–1^ NIV, the roots of both transgenic lines were significantly longer than those of Col-0 (Fig. 3D; Supplementary Fig. S3). Therefore, transgenic *A. thaliana* expressing *HvUGT13248* show increased resistance to high concentrations of NIV.

### Transgenic wheat expressing *HvUGT13248* exhibits type II resistance to NIV-producing *F. graminearum*

**Fig. 4.**
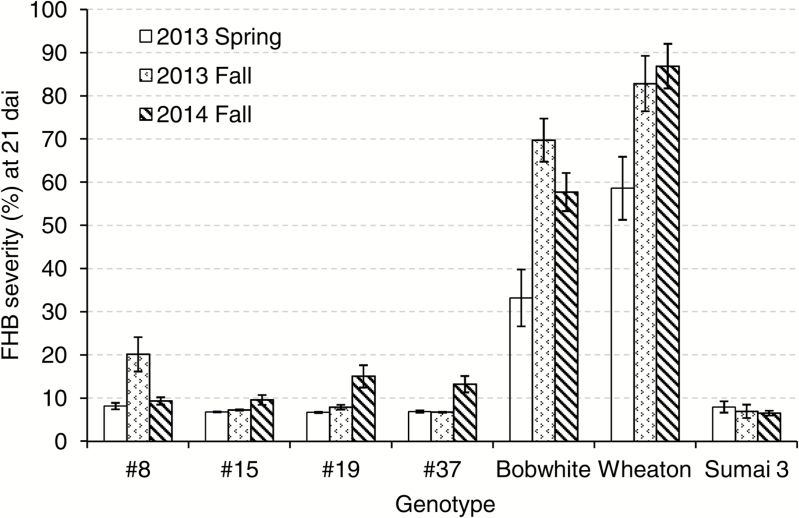
FHB severity of transgenic wheat expressing *HvUGT13248* at 21 d after point inoculation with NIV-producing *F. graminearum* strain #02-15 in three greenhouse trials. Lines #8, #15, #19, and #37 were transgenic wheat expressing *HvUGT13248*, and ‘Bobwhite’ was the non-transformed control. ‘Wheaton’ was the susceptible check and ‘Sumai 3’ was the resistant check.

To test NIV resistance in transgenic wheat expressing *HvUGT13248*, we performed point inoculation assays with a NIV-producing *F. graminearum* strain (Gale *et al.*, 2011). Transgenic wheat carrying *HvUGT13248* (independent transgenic lines *#*8, #15, #19, and #37 previously described in Li *et al.*, 2015), ‘Bobwhite’, ‘Sumai 3’, and ‘Wheaton’ were evaluated for type II resistance. We repeated this disease screen in the greenhouse three times and, for each screen, 20–32 plants of each transgenic event were grown. The susceptible check ‘Wheaton’ exhibited FHB severity levels that ranged between 58.6 ± 7.3% and 86.9 ± 5.1%, while the resistant check ‘Sumai 3’ exhibited FHB severity levels that ranged between 6.5 ± 0.6% and 8.0 ± 1.3%, indicating that the environments for FHB severity screening were successful and discriminative (Fig. 4; Supplementary Table S1). The checks also demonstrate differences in FHB severity between the trials, which is a frequent observation when assaying FHB severity (e.g. Okubara *et al.*, 2002; Li *et al.*, 2015). The transgenic lines significantly reduced FHB severity compared with the non-transformed ‘Bobwhite’ control (Fig. 4; Supplementary Table S1). For the three greenhouse trials, transgenic line #8 showed FHB severity of 8.2–20.2%, #15 showed 6.8–9.6% severity, #19 showed 6.7–15.1% severity, and #37 showed 6.8–13.2% severity. All four transgenic lines exhibited a significant reduction of FHB severity relative to ‘Bobwhite’, ranging from 71.1% to 90.3%. Three of the transgenic lines (#15, #19, and #37) showed FHB severity in more than one trial at levels similar to the resistant line ‘Sumai 3’. NIV and ergosterol contents were also measured on whole spikes by GC-MS during the 2013 greenhouse trial (Table 2). All four transgenic lines accumulated significantly less NIV and ergosterol compared with ‘Bobwhite’. DON did not accumulate to detectable levels in the transgenic lines or ‘Sumai 3’; however, in ‘Bobwhite’ and ‘Wheaton’, we observed a small amount of DON accumulation. These results indicate that the NIV-producing strain also produces a trace amount of DON, and when inoculated on genotypes (e.g. ‘Wheaton’ and ‘Bobwhite’) that have less capacity to detoxify trichothecenes, a small amount of DON is detected. Taken together, transgenic wheat expressing *HvUGT1348* exhibited high levels of type II resistance to a NIV-producing *F. graminearum* strain and lowered NIV content.

**Table 2.** Trichothecene accumulation and ergosterol content in NIV-producing *F. graminearum*-inoculated wheat spikes

**Genotype** ^***a***^	**Ergosterol (mg l** ^**–1**^)	**NIV (mg l** ^**–1**^)	**DON (mg l** ^**–1**^)
#8	5.38 ± 1.58***	4.70 ± 0.50***	ND^*b*^
#15	1.37 ± 0.50***	2.00 ± 0.31***	ND
#19	1.85 ± 0.05***	3.03 ± 0.58***	ND
#37	1.08 ± 0.20***	1.80 ± 0.06***	ND
Bobwhite	49.53 ± 6.95	29.57 ± 5.82	0.28 ± 0.06
Wheaton	54.88 ± 4.23	47.43 ± 2.16	0.29 ± 0.02
Sumai 3	0.90 ± 0.02	0.58 ± 0.02	ND

Values provided are the means ±SE.

^*a*^ #8, #15, #19, and #37 were transgenic lines, and ‘Bobwhite’ was the non-transformed control. ‘Sumai 3’ was the resistant check and ‘Wheaton’ was the susceptible check.

^*b*^ ND, not detected (<0.05 mg l^–1^).

*** indicate signiﬁcance at the 0.001 level compared with the non-transformed ‘Bobwhite’ control (Student’s *t*-test).

### Increased NIV to NIV3G conversion in transgenic wheat

To determine if the enhanced type II resistance conferred by *HvUGT13248* was indeed due to increased glycosylation, we monitored the NIV and NIV3G concentrations in transgenic line #19 and the ‘Bobwhite’ non-transgenic control from 0 d to 14 d after NIV application. Two central spikelets on the main spike of transgenic line #19 and non-transformed ‘Bobwhite’ were inoculated with NIV at 40 µg per spikelet (80 µg or 256.41 nmol NIV per spike). Treated spikelets together with the connecting rachis tissue were collected at 10 time points: 0, 2, 6, 12, 24, 36, 48, 72, and 96 h, and 14 d after NIV application. NIV and NIV3G concentrations were measured by LC-MS/MS.

**Fig. 5.**
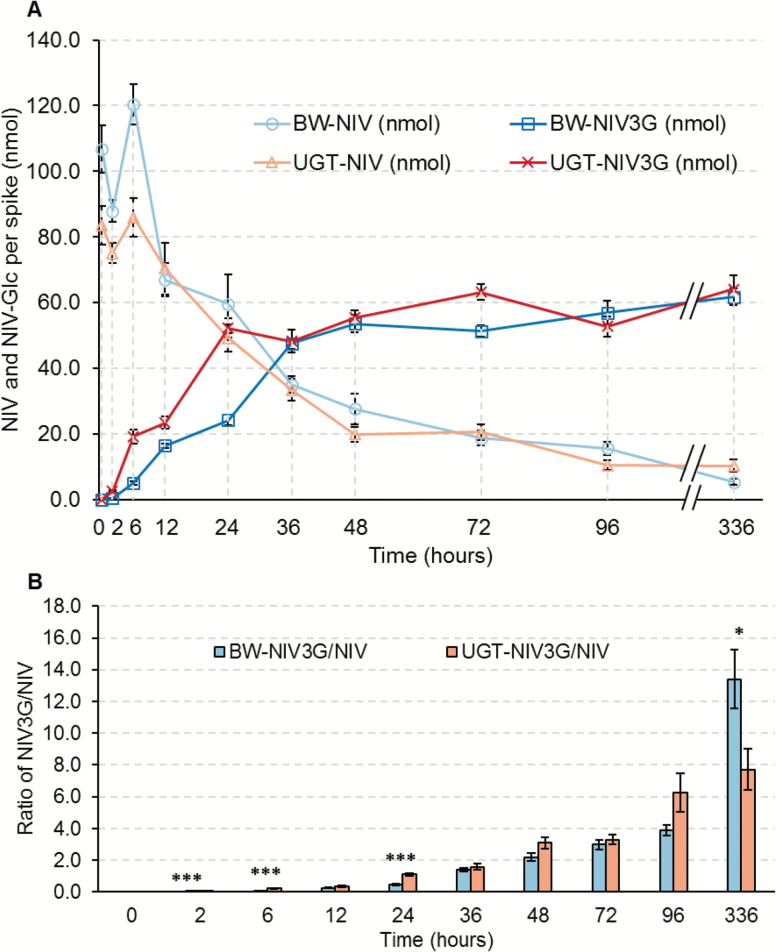
*HvUGT13248* promotes NIV to NIV3G conjugation in transgenic wheat. (A) NIV and NIV3G concentrations in ‘Bobwhite’ (BW) and transgenic line #19 at 0, 2, 6, 12, 24, 36, 48, 72, 96, and 336 h after NIV treatment. ‘BW-NIV’ and ‘UGT-NIV’ are the NIV content in BW or transgenic event #19 at each time point, respectively. ‘BW-NIV3G’ and ‘UGT-NIV3G’ are the NIV3G content in BW or transgenic line #19 at each time point, respectively. (B) Fold change of the molar ratio of NIV3G to NIV concentrations in BW and transgenic line #19 at each time point.

NIV concentration decreased and NIV3G concentration increased in both transgenic and non-transformed wheat (Fig. 5; Supplementary Table S2), indicating that both genotypes possess NIV-glycosyltransferase activity. However, the conversion was faster in transgenic line #19 than in ‘Bobwhite’ at early time points. Before 6 h after NIV injection, NIV concentrations in line #19 were significantly lower than in ‘Bobwhite’, while NIV3G contents were significantly higher than in ‘Bobwhite’. NIV3G concentrations continued to be significantly higher in line #19 at 12 h and 24 h after treatment. Average NIV3G/NIV ratios in the transgenic lines were significantly higher than in ‘Bobwhite’ during the first 24 h after NIV application, except for the 12 h time point. After 36 h, the differences between transgenic and non-transformed lines were not significant, indicating that the greatest impact of *HvUGT13248* overexpression on NIV to NIV3G conversion is at early time points.

## Discussion

### The *HvUGT13248* gene product can metabolize different trichothecene toxins

Family 1 UGTs comprise a large family of genes (Caputi *et al.*, 2012). UGTs play important roles in diverse biological processes (Ross *et al.*, 2001) and UGT genes seem to evolve rapidly by amplification and gene death; thus, it is non-trivial to identify true orthologs in related plant genomes (Schweiger *et al.*, 2013). Moreover, individual plant UGTs may accept more than one substrate. For example, *A. thaliana AtUGT73C5*, the first UGT described to inactivate DON (Poppenberger *et al.*, 2003), can also glycosylate brassinosteroids (Poppenberger *et al.*, 2005), the structurally unrelated *Fusarium* toxin zearalenone (Poppenberger 2003), and regioselectively also quercetin at certain hydroxyl groups (Lim *et al.*, 2004). Surprisingly, despite this broad activity towards multiple structurally unrelated substrates, *AtUGT73C5* does not confer NIV resistance (Fig. 1), even though only the presence of the C4-OH distinguishes NIV from DON. In contrast, the rice OsUGT79 can metabolize both DON and NIV, but not T-2 toxin (Wetterhorn *et al.*, 2016). Likewise, the barley *HvUGT13248* also efficiently metabolizes NIV and DON. Our NMR results show that the mode of NIV detoxification is analogous to that of DON, by exclusive formation of NIV3G. The HvUGT13248 enzyme has higher affinity (*K*_m_ value in Table 1) for NIV and about five times more efficiency (*k*_cat_/*K*_m_ in Table 1) than with DON as the substrate.

Inactivation of the *Brachypodium* gene *Bradi5g0330*0, which is most similar to *HvUGT13248*, causes reduced resistance to *Fusarium* infection (Pasquet *et al.*, 2016). Assuming that HvUGT13248 is also a highly relevant detoxification enzyme in barley, the observation of a much lower affinity of the enzyme towards DON than NIV suggests that the switch from the ancestral NIV production to DON production in *Fusarium* by mutation of *TRI13* (Brown *et al.*, 2002; Lee *et al.*, 2002) might have allowed DON partially to escape detoxification, providing a selective advantage to DON producers on barley. DON and NIV chemotype strains co-exist in the field and seem to be maintained by balancing selection (Ward *et al.*, 2002). We have also identified a glucosyltransferase from *Brachypodium* which confers resistance to NIV but is nearly inactive with DON (G. Wiesenberger *et al*., unpublished results). Therefore, it is conceivable that differences in the detoxification capacity in various host plants exist, making production of either DON or NIV advantageous. Interestingly, NIV production has been described to be a virulence factor on maize (Maier *et al.*, 2006), while disruption of DON production had little effect, which could be explained by high DON detoxification capability. Yet, DON producers are in general also predominant in maize grown in Europe, North America, and China (Qiu and Shi, 2014; Kuhnem *et al.*, 2015; Pasquali *et al.*, 2016), while in South American-grown maize high frequencies of NIV-producing *F. meridionale* and *F. boothii* were found (Sampietro *et al.*, 2012). Interestingly, strains have been reported that were genotyped as NIV producers, but produced up to 20% DON besides NIV, indicating that not only is complete loss of function by disrupting the *TRI13* coding region possible, but potentially also down-regulation of the *TRI1* expression level. Overall, it seems likely that genotypic variation within different crop species with respect to detoxification capacity is high, and drawing conclusions based on one or a few cultivars is premature. Currently, information on the detoxification capability of different cultivars is lacking in most crop plants infected by *Fusarium* ssp.

### Higher intrinsic plant resistance to NIV than to DON

Overexpression of *HvUGT13248* provided resistance to NIV in *A. thaliana*, alleviating the detrimental effect of the toxin on root length at 100 mg l^–1^ NIV (Fig. 3). However, compared with similar assays with DON (Shin *et al.*, 2012), we found that the minimal concentration required to observe root inhibition in the wild type Col-0 is dramatically higher for NIV (20 mg l^–1^ no inhibition, Fig. 3B) than for DON (0.5 mg l^–1^, Shin *et al.*, 2012). This is consistent with a previous report of relatively lower phytotoxicity of NIV compared with DON in an *A. thaliana* leaf assay (Desjardins *et al.*, 2007). Similar results have been previously reported in wheat (Shimada and Otani, 1990), which showed severe wheat root growth inhibition by DON, while observing no such differences for NIV at the same concentrations.

In contrast, NIV has higher cytotoxicity than DON when applied orally to experimental animals, and is ~1.5- to 1.7-fold more toxic to Caco-2 human cells (Alassane-Kpembi *et al.*, 2013). NIV was also shown to have greater impact than DON on the pig intestinal mucosa, both *in vitro* and *in vivo* (Cheat *et al.*, 2015). Our data obtained with the *in vitro* translation systems (Fig. 2; Supplementary Fig. S2) show that this discrepancy between plants and animals is not due to differences at the ribosomal target. NIV is slightly more inhibitory than DON for both rabbit and plant ribosomes. The difference in toxicity in plants may be in part at the level of uptake or drug efflux (e.g. substrate specificity for ABC transporters). Yet, it seems likely that the ability to glycosylate these toxins plays an important role. Interestingly, there is also a difference in animals. While DON is converted into glucuronides in pigs (and other experimental animals and humans) and *in vitro* by different UDP-glucuronosyltransferases (Maul *et al.*, 2015), no evidence for formation of NIV-glucuronide was detected in a pig feeding study (Hedman *et al.*, 1997) and in mice treated with NIV (Poapolathep *et al.*, 2003), which might be a reason for the higher toxicity of NIV in animals.

Our data suggest that the higher basal resistance of wheat to NIV compared with DON directly correlates with the higher levels of NIV3G found in the susceptible wheat cultivar ‘Bobwhite’ compared with D3G following treatment with the respective toxin. Apparently, wheat has endogenous UGTs to inactivate NIV but a much lower capacity to detoxify DON. Constitutive expression of *HvUGT13248* clearly increased resistance to a NIV-producing *F. graminearum*, although the capacity to detoxify externally applied NIV was high in both parental and transgenic wheat. The transgenic lines converted 62.6% of the administered NIV into the glucoside 24 h after treatment, while ‘Bobwhite’ metabolized only 22.7%. By 36 h after NIV treatment, transgenic wheat converted 57.7% of the toxin to NIV3G, while non-transformed ‘Bobwhite’ converted 44.5% (Fig. 5; Supplementary Table S2). In comparison, in a similar DON treatment experiment, ‘Bobwhite’ metabolized only 2.2% DON to D3G 24 h after treatment (Li *et al.*, 2015).

NIV-producing *F. graminearum* inoculated on transgenic wheat resulted in a lower NIV content (and reduced fungal biomass) and a higher NIV3G to NIV ratio compared with non-transformed ‘Bobwhite’. One can expect that this is also the case in natural infection with NIV producers in the field. NIV3G is a neglected ‘masked mycotoxin’ of unknown toxicological relevance. Yet, based on results of the reticulocyte lysates and experience from D3G in mice and pigs, resorbed NIV3G itself will not be toxic for mammalian ribosomes, and the back-conversion of NIV3G to NIV by bacterial glucosidases should also take place rather late in the intestinal tract as demonstrated for D3G (Nagl *et al.*, 2014), so that most of the released NIV should not be resorbed but excreted via feces (Gratz *et al.*, 2016). The lack of an analytical standard for NIV3G and the large amounts needed for toxicological studies have to date prevented research on this issue. So far only small amounts of NIV3G have been purified from NIV-contaminated wheat (9 mg from 12 kg of starting material; Yoshinari *et al.*, 2014). We showed that NIV3G can be efficiently synthesized enzymatically *in vitro* with the rice enzyme OsUGT79. As the reaction can be driven to completion by cofactor recycling (as shown for D3G; Michlmayr *et al.*, 2015) and the reaction mix is much less complex, purification from this source is much easier. The described method can easily provide gram amounts of NIV3G for animal feeding studies, which will allow experimental testing of our predictions.

### Rapid trichothecene detoxification is key to FHB resistance

The interaction between the fungal pathogen and the plant host is very dynamic. Trichothecenes play an important role in the disease spread during FHB development (Bai *et al.*, 2002), and reducing trichothecenes is important to type II resistance (Li *et al.*, 2015). Gene expression profiles have shown that *F. graminearum* induces trichothecene biosynthesis genes as early as 48 h after germination in wheat (Lysøe *et al.*, 2011), and Boenisch and Schäfer (2013) reported similar timing based on microscopic examination of reporter genes. In barley and *Brachypodium*, the DON-inactivating UGTs are highly inducible by the toxin (Gardiner *et al.*, 2010; Schweiger *et al.*, 2013). Upon perception of the toxin, it depends on how rapidly the already partially inhibited ribosome can translate the induced UGT transcript into an active detoxification enzyme. If the toxin can be efficiently neutralized, the plant can contain the pathogen, limiting its spread. If the toxin diffusing ahead of the infection zone blocks or at least strongly reduces or severely delays translation, the plant is susceptible and the pathogen can spread throughout the spike. Consistent with this model, transgenic wheat constitutively overexpressing *HvUGT13248* efficiently converted NIV to NIV3G, leading to high levels of resistance to disease spread after inoculation with a NIV-producing *F. graminearum* (Fig. 4; Table 2; Supplementary Table S1). The ability of the susceptible ‘Bobwhite’ and the transgenic resistant line to convert comparable amounts of NIV to NIV3G at 36 h after administering the toxin and later suggests that resistance against NIV-producing chemotypes is constituted at earlier time points.

With constitutive overexpression, transgenic wheat expressing *HvUGT13248* provides resistance to both DON and NIV and therefore does not seem to be at risk of being easily overcome by a shift in chemotype composition, and thus is an excellent candidate gene for FHB control also in regions of the world where NIV-producing species are highly prevalent.

## Supplementary data

Supplementary data are available at *JXB* online.

**Fig. S1.** NMR data of NIV-3-*O*-β-d-glucoside.

**Fig. S2.** NIV-3-glucoside is less inhibitory than NIV for rabbit reticulocyte ribosomes.

**Fig. S3**. Root growth of Col-0 wild-type and transgenic *A. thaliana* expressing *HvUGT13248* on half-strength MS medium containing 0 mg l^–1^ NIV at 7 d and 100 mg l^–1^ NIV at 14 d after germination. Scale bars=2 cm.

**Table S1.** Summary of transgenic wheat expressing *HvUGT13248* in greenhouse point-inoculation tests with the NIV-producing *F. graminearum* strain.

**Table S2.** HvUGT13248 converts NIV to NIV3G faster in transgenic wheat than in non-transformed ‘Bobwhite’.

## Supplementary Material

supplementary_figures_S1_S3_Table_S1_S2

## Abbreviations:

D3G: DON-3-*O*-β-d-glucoside
DON: deoxynivalenol
FHB: Fusarium Head Blight
NIV: nivalenol
NIV3G: NIV-3-*O*-β-d-glucoside
UGT: UDP-glycosyltransferase.
