# Supplementary material for: A barley UDP-glucosyltransferase inactivates nivalenol and provides Fusarium Head Blight resistance in transgenic wheat
Source: J Exp Bot. 2017 Apr 12;68(9):2187–97. doi: 10.1093/jxb/erx109 (PMC5447872; doi:10.1093/jxb/erx109)
Supplement: supplementary_figures_S1_S3_Table_S1_S2 [file erx109_suppl_supplementary_figures_S1_S3_Table_S1_S2.pdf]

A barley UDP-glucosyltransferase inactivates nivalenol and provides Fusarium Head Blight resistance in transgenic wheat

Xin Li , Herbert Michlmayr, Wolfgang Schweiger, Alexandra Malachova, Sanghyun Shin, Yadong Huang, Yanhong Dong, Gerlinde Wiesenberger, Susan McCormick, Marc Lemmens, Philipp Fruhmann, Christian Hametner, Franz Berthiller, Gerhard Adam, and Gary Muehlbauer

Supplementary File

Fig. S1. NMR data of NIV-3-*O*-β-D-glucoside. ^{1}H, ^{13}C and correlated spectra revealing NIV3G as the only product of NIV detoxification by OsUGT79 and HvUGT13248. All chemical shifts are displayed on the x-axis and are given in ppm relative to tetramethylsilane. The calibration was done using residual solvent signals methanol-*d4*). Multiplicities are abbreviated as s (singlet), d (doublet), t (triplet), q (quartet) and b (broad signal).

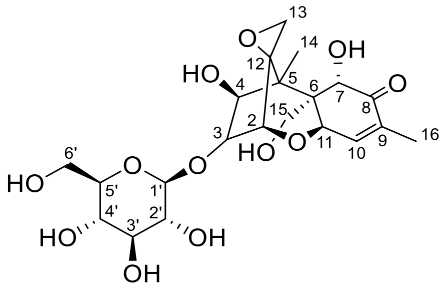

a) ^{1}H spectrum of NIV-3-β-D-glucoside

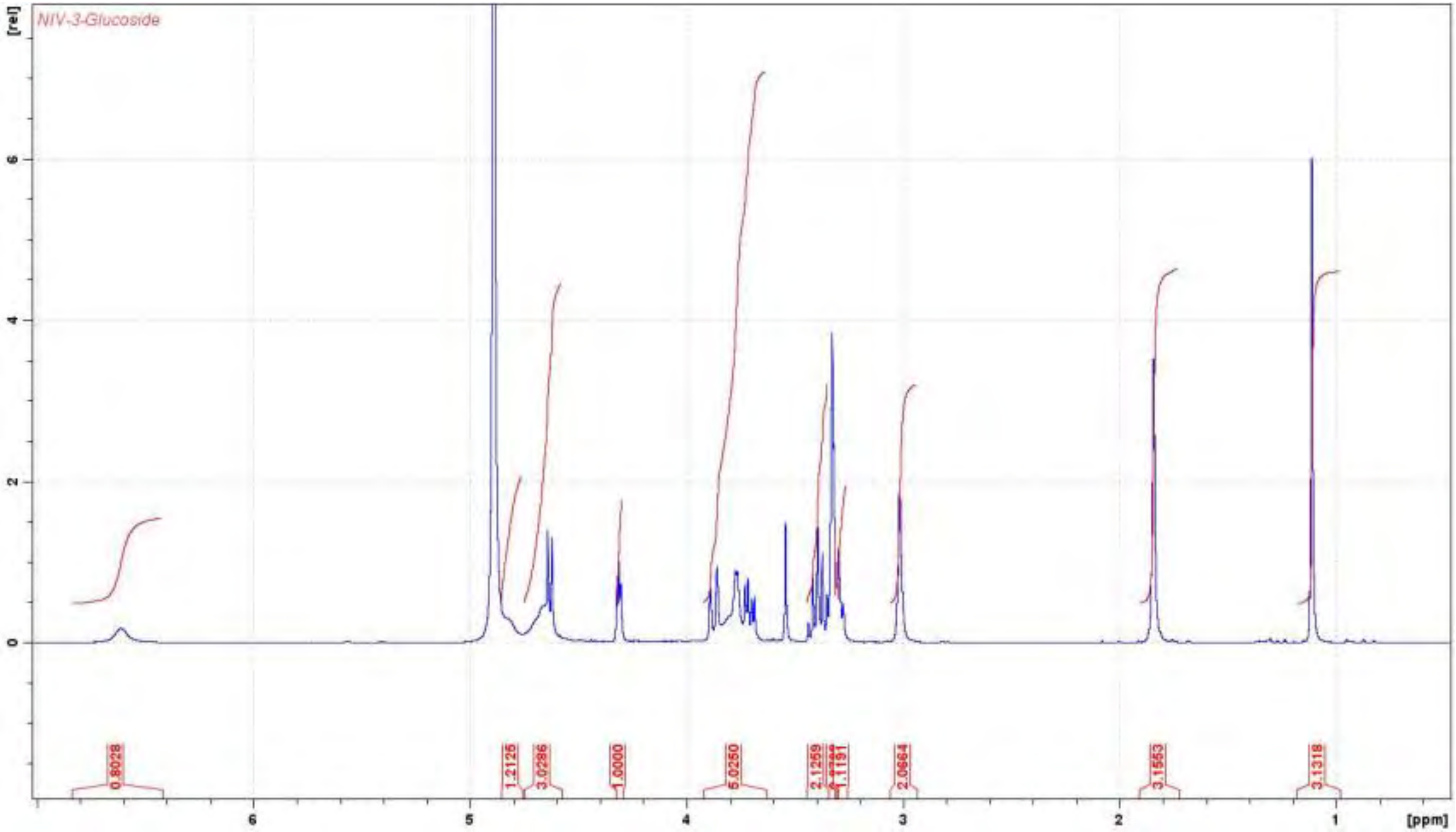

^{1}H NMR (400 MHz, methanol-d_4) δ 6.61 (b, 1H), 4.82 (b, 1H), 4.69 (b, 1H), 4.66 (b, 1H), 4.63 (d, *J*=7.6 Hz, 1H), 4.31 (dd, *J*=4.5, 3.7 Hz, 1H), 3.88 (dd, *J*=12.0, 2.1 Hz, 1H), 3.77 (d, *J*=4.1 Hz, 1H), 3.71 (dd, *J*=12.1, 5.0 Hz, 1H), 3.70 – 3.90 (m, 2H), 3.40 (t, *J*=7.9 Hz, 1H), 3.37 (t, *J*=8.5 Hz, 1H), 3.31 (b, 1H), 3.29 (b, 1H), 3.02 (b, 2H), 1.84 (s, 3H), 1.11 (s, 3H)

b) ^{13}C spectrum of NIV-3-β-D-glucoside

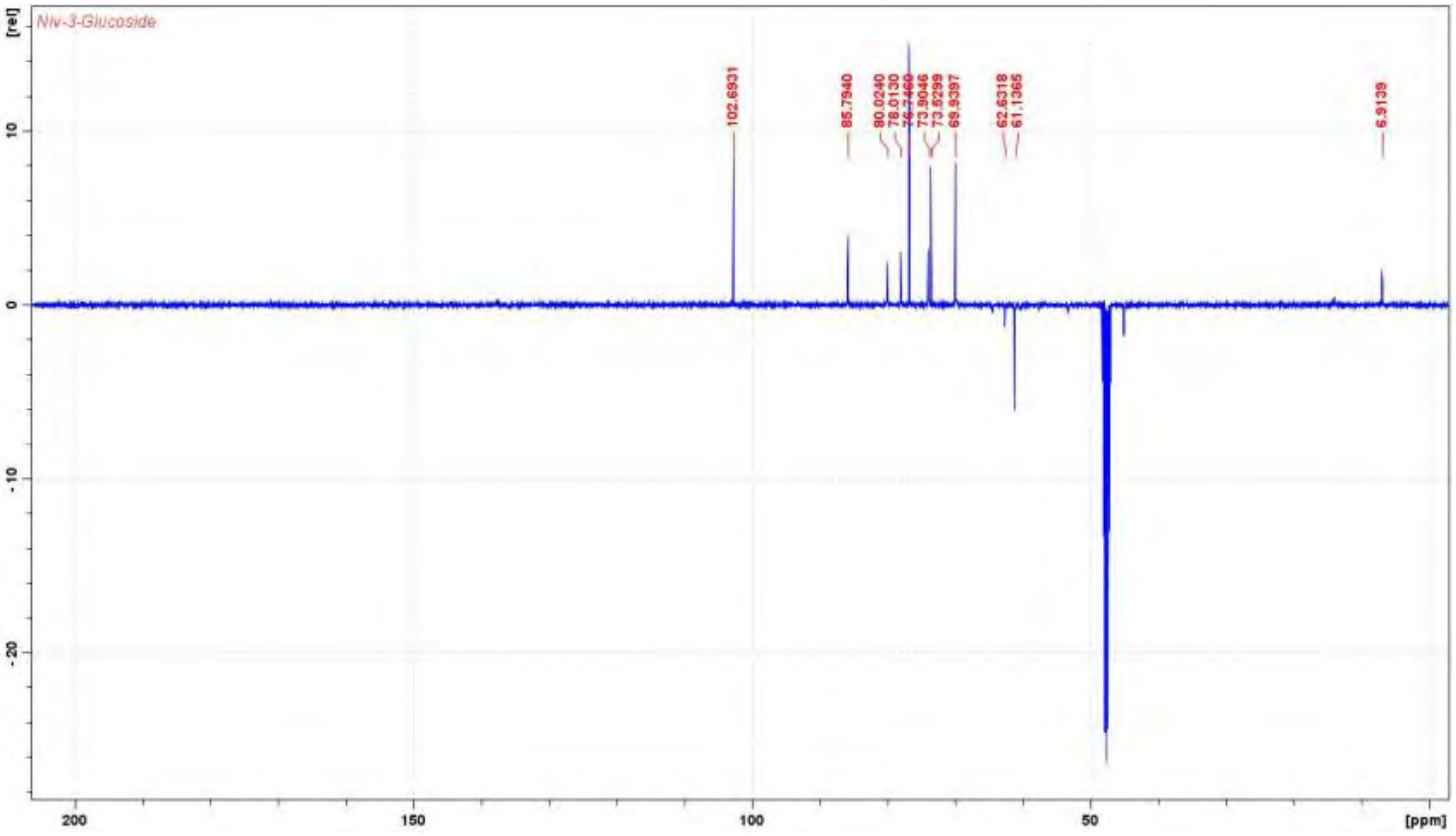

^{13}C NMR (100 MHz, methanol-d_4) δ 200.1 (s, 1C), 137.5 (d, 1C), 136.1 (s, 1C), 102.7 (d, 1C), 85.8 (d, 1C), 80.0 (d, 1C), 78.0 (d, 1C), 76.7 (d, 2C), 74.0 (d, 1C), 73.5 (d, 1C), 69.9 (d, 1C), 69.6 (d, 1C), 64.5 (s, 1C), 61.1 (t, 1C), 60.0 (t, 1C), 53.3 (s, 1C), 48.8 (s, 1C), 45.1 (t, 1C), 13.8 (q, 1C), 6.9 (q, 1C)

c) H-H-COSY spectrum of NIV-3-β-D-glucoside

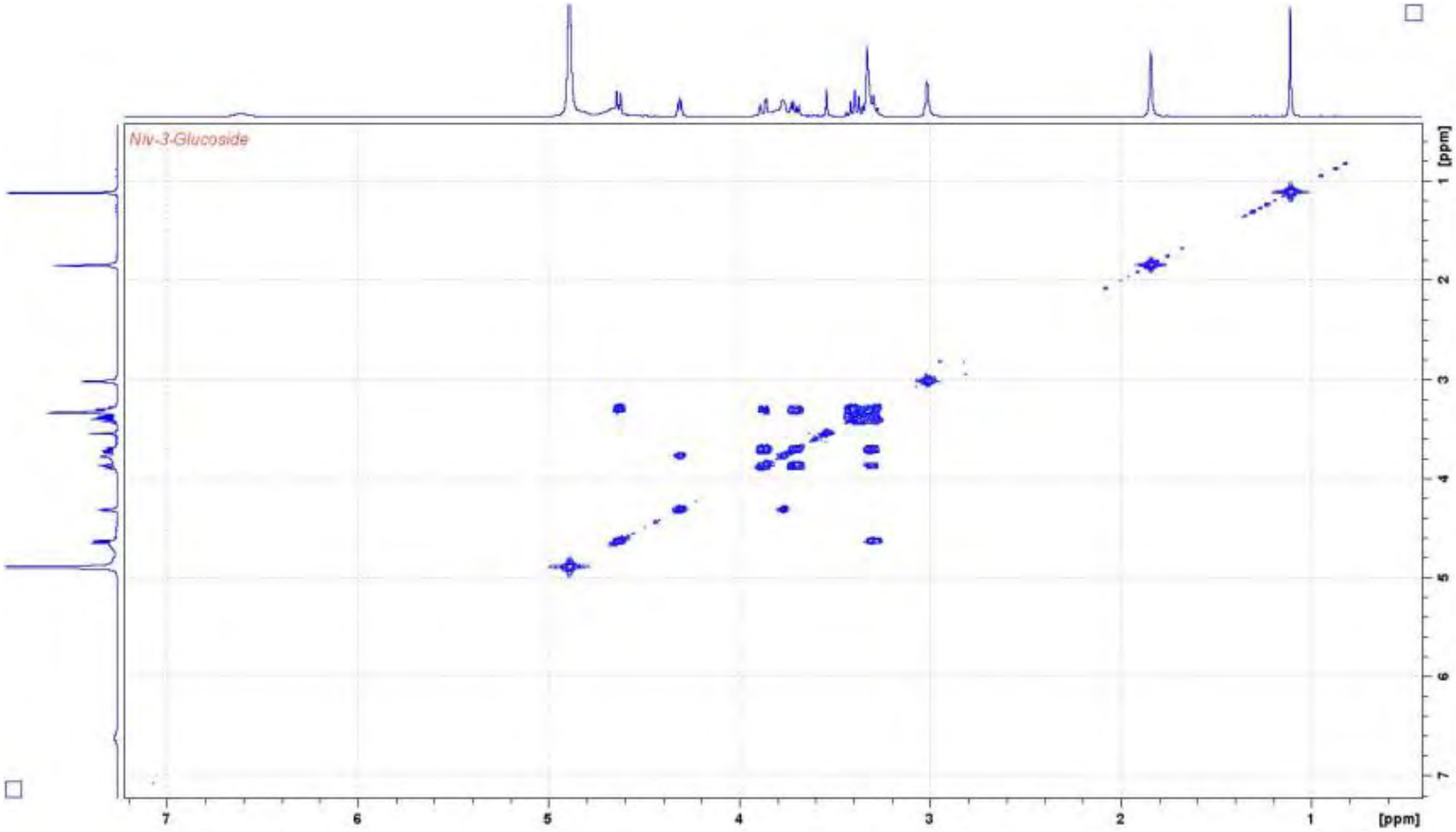

d) HSQC spectrum of NIV-3-β-D-glucoside with ^{1}H on the x-axis and ^{13}C on the y-axis

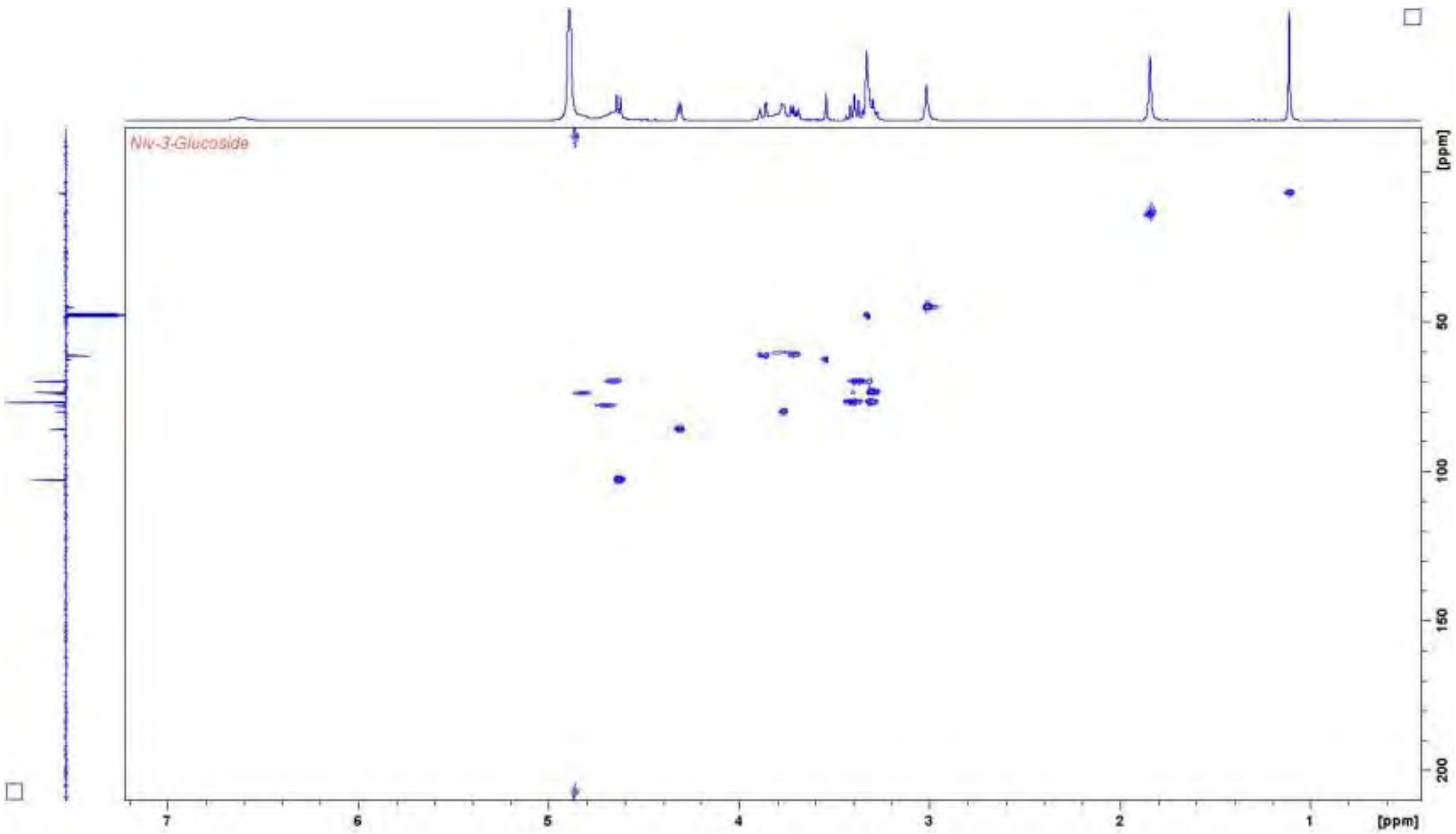

e) HMBC spectrum of NIV-3-β-D-glucoside with ^{1}H on the x-axis and ^{13}C on the y-axis. The arrows indicate the connection between position 3 of DON and position 1’ of glucose - red arrow = 85.8/4.63 ppm; blue arrow = 102.7/4.31 ppm.

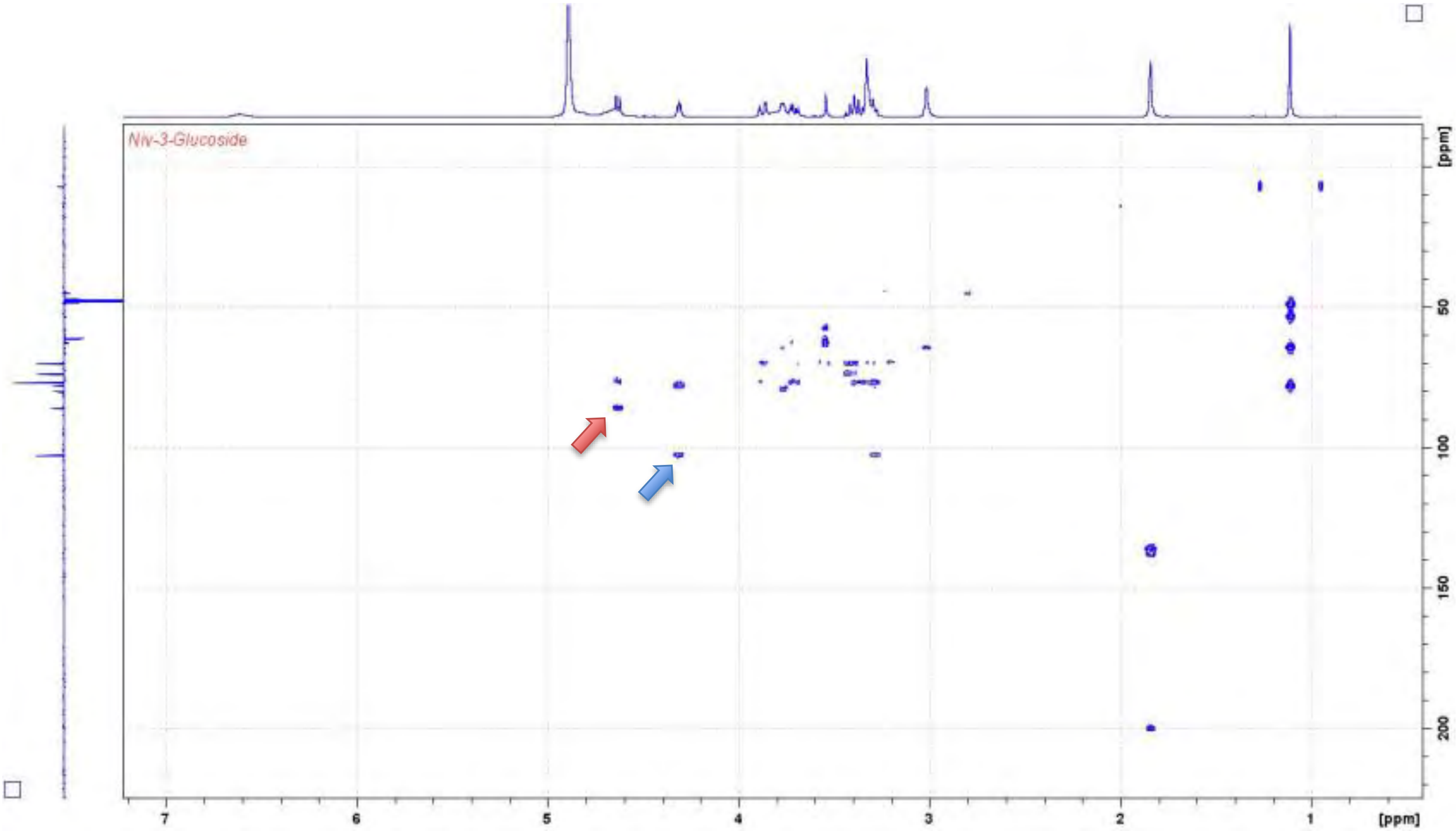
Niv-3-Glucoside
[ppm]
50
100
150
200
7
6
5
4
3
2
1
[ppm]

Fig. S2. NIV-3-glucoside is less inhibitory than NIV for rabbit reticulocyte ribosomes.

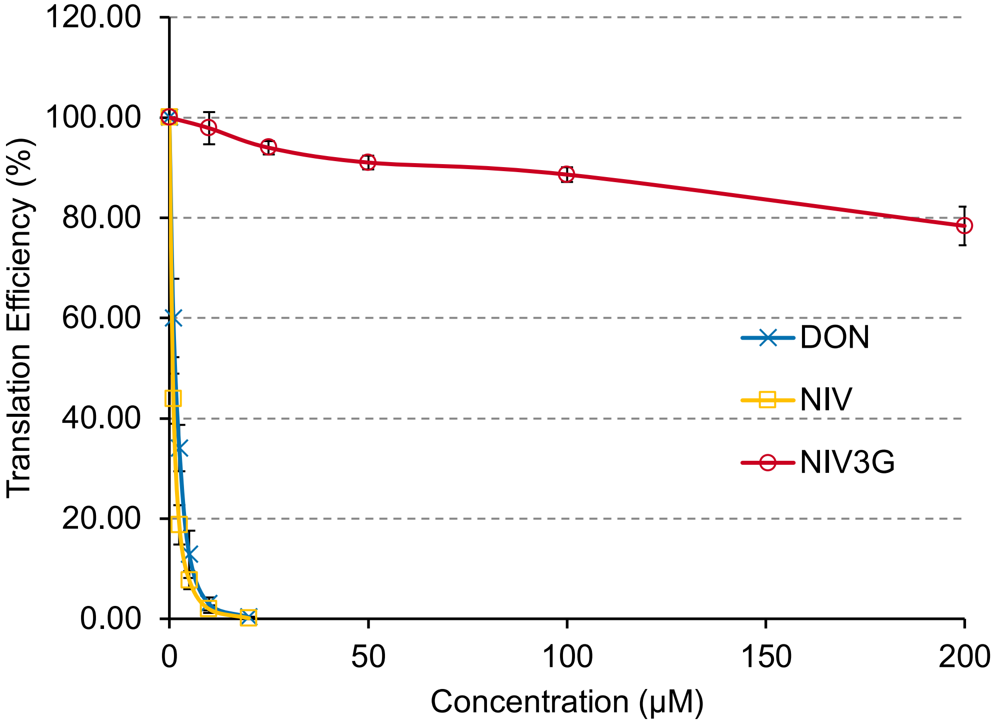

Fig. S3. Root growth of Col-0 wildtype and transgenic *A. thaliana* expressing *HvUGT13248* on half strength MS medium containing 0 mg L^{-1} NIV at 7 days and 100 mg L^{-1} NIV at 14 days after germination. Scale bars = 2 cm.

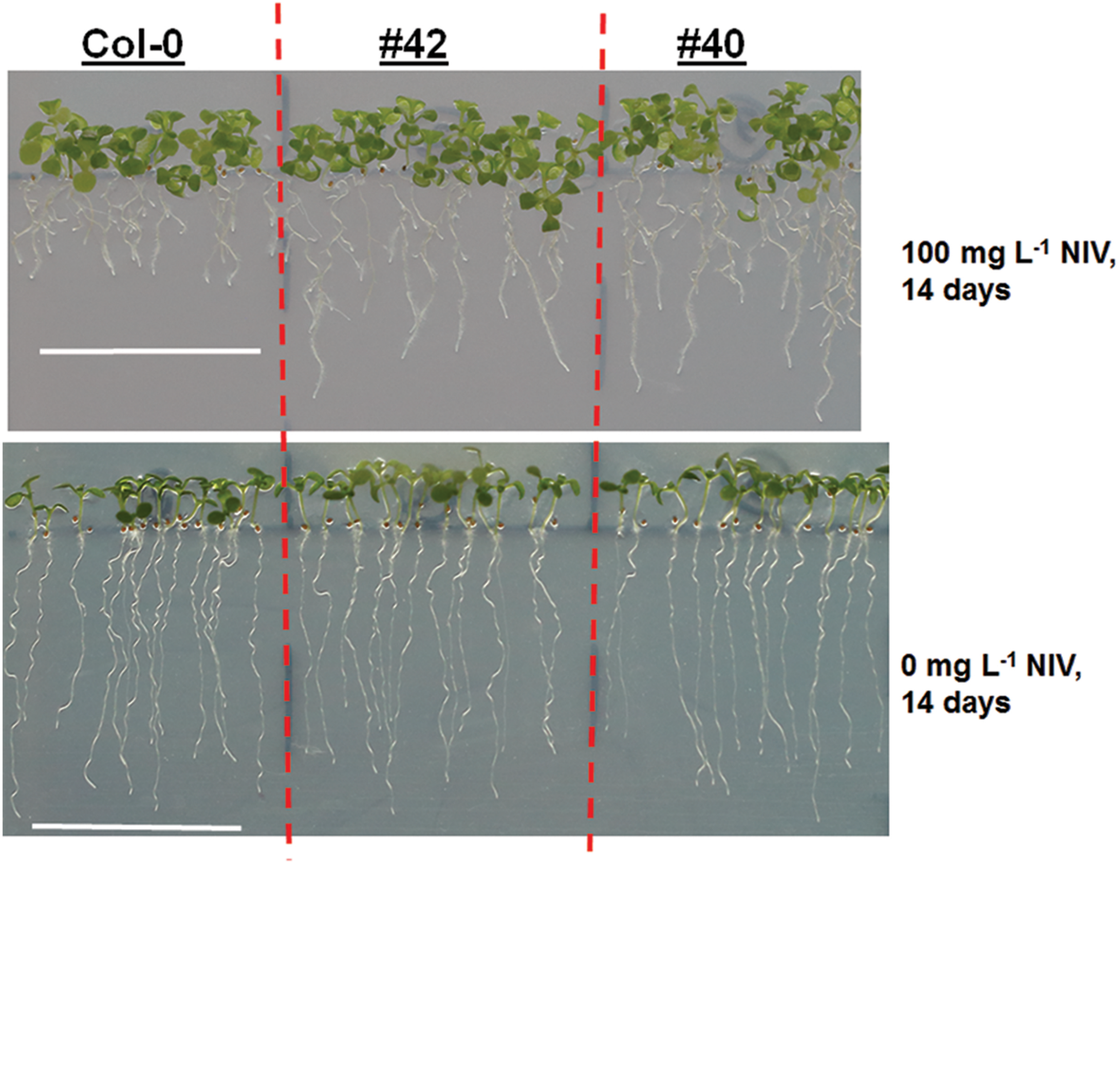

Table S1. Summary of transgenic wheat expressing *HvUGT13248* in greenhouse point-inoculation tests with NIV-producing *F. graminearum* strain.

	2013 Spring			2013 Fall			2014 Fall		
**Genotype[a]**	**No.[b]**	**Sev.[c] (%)**	**Red.[d] (%)**	**No.**	**Sev. (%)**	**Red. (%)**	**No.**	**Sev. (%)**	**Red. (%)**
#8	31	8.2 ± 0.8***	75.4	27	20.2 ± 4.0***	71.1	16	9.4 ± 0.9***	83.8
#15	32	6.8 ± 0.1***	79.5	27	7.3 ± 0.1***	89.6	14	9.6 ± 1.1***	83.4
#19	20	6.7 ± 0.2***	79.9	18	7.9 ± 0.5***	88.6	14	15.1 ± 2.6***	73.9
#37	31	6.9 ± 0.3***	79.3	27	6.8 ± 0.1***	90.3	16	13.2 ± 1.9***	77.1
Bobwhite	22	33.2 ± 6.6	NA[e]	25	69.8 ± 5.0	NA	16	57.7 ± 4.4	NA
Wheaton	23	58.6 ± 7.3	NA	20	82.9 ± 6.4	NA	15	86.9 ± 5.2	NA
Sumai 3	22	8.0 ± 1.3	NA	18	7.0 ± 1.6	NA	18	6.5 ± 0.6	NA

[a]Events #8, #15, #19 and #37 were transgenic lines, and ‘Bobwhite’ was the non-transformed control. ‘Sumai 3’ was the resistant check, and ‘Wheaton’ was the susceptible check.

Values provided are the means ± standard error.

*, ** and *** indicate significance at the 0.05, 0.01, and 0.001 levels compared with the non-transformed ‘Bobwhite’ control (Student’s t test).

[b]No.: number of plants examined in the disease screen

[c]Sev.: FHB severity shown as the percentage of symptomatic spikelets in the inoculated spikes

[d]Red.: percent disease reduction rate as compared to the corresponding non-transformed ‘Bobwhite’ control.

[e]NA: not applicable.

Table S2. *HvUGT13248* converts NIV to NIV3G faster in transgenic wheat than in non-transformed 'Bobwhite'. Numbers shown are mean value ± standard error. *, ** and *** indicate significance at the 0.05, 0.01, and 0.001 levels compared with the non-transformed 'Bobwhite' control (Student's t test).

	**Non-transformed 'Bobwhite'**				***HvUGT13248*-#19 transgenic wheat**			
**Time[a] (h)**	**NIV (nmol)**	**NIV3G (nmol)**	**NIV3G/ NIV[b]**	**NIV+NIV3G[c] (nmol)**	**NIV (nmol)**	**NIV3G (nmol)**	**NIV3G/ NIV**	**NIV+NIV3G (nmol)**
0	106.69 ± 7.17	0	0	106.69 ± 7.17	83.57 ± 5.82*	0	0	83.57 ± 5.82*
2	87.89 ± 3.40	0.44 ± 0.11	0.01 ± 0	88.39 ± 3.39	75.06 ± 3.13*	2.52 ± 0.22***	0.03 ± 0***	77.58 ± 3.32*
6	120.45 ± 6.18	5.02 ± 0.44	0.04 ± 0	125.47 ± 6.20	85.96 ± 5.85**	19.20 ± 2.11***	0.22 ± 0.02***	105.16 ± 7.20
12	66.92 ± 5.05	16.37 ± 0.77	0.26 ± 0.02	83.29 ± 5.22	70.35 ± 7.90	23.41 ± 1.90**	0.36 ± 0.05	93.75 ± 7.91
24	59.55 ± 8.95	24.24 ± 1.57	0.45 ± 0.04	83.79 ± 9.64	49.13 ± 4.13	52.28 ± 2.97***	1.11 ± 0.09***	101.41 ± 5.84
36	35.04 ± 2.55	47.53 ± 1.60	1.40 ± 0.09	82.56 ± 3.26	33.44 ± 3.33	48.19 ± 3.44	1.59 ± 0.20	81.63 ± 2.60
48	27.64 ± 4.62	53.62 ± 2.76	2.20 ± 0.24	81.26 ± 6.64	19.84 ± 2.29	55.47 ± 2.19	3.10 ± 0.36	75.31 ± 2.33
72	18.76 ± 2.22	51.34 ± 1.72	2.99 ± 0.32	70.10 ± 2.83	20.52 ± 2.42	63.18 ± 2.49**	3.31 ± 0.29	74.40 ± 10.09*
96	15.58 ± 1.92	56.89 ± 3.61	3.91 ± 0.33	64.42 ± 9.23	10.45 ± 1.41*	52.71 ± 3.13	6.24 ± 1.21	63.16 ± 3.31
336	5.41 ± 0.89	61.75 ± 2.43	13.40 ± 1.87	59.70 ± 7.87	10.35 ± 1.83*	64.12 ± 4.25	7.72 ± 1.30*	66.20 ± 9.47

[a]Time after NIV treatment when samples were collected.

[b]Ratio of NIV3G over NIV at each time point in each genotype.

[c]Sum of NIV3G and NIV at each time point in each genotype.
